# Comparative Transcriptome Analysis for Understanding Predator-Induced Polyphenism in the Water Flea *Daphnia pulex*

**DOI:** 10.3390/ijms19072110

**Published:** 2018-07-20

**Authors:** Haein An, Thinh Dinh Do, Gila Jung, Mustafa Zafer Karagozlu, Chang-Bae Kim

**Affiliations:** 1 Department of Biotechnology, Sangmyung University, Seoul 03016, Korea; haein0415@gmail.com (H.A.); deepblue.th@gmail.com (T.D.D.); gilajung@gmail.com (G.J.); zaferka@gmail.com (M.Z.K.); 2 E-biogen Company, Seoul 07282, Korea

**Keywords:** *Daphnia pulex*, multiple developmental stages, inducible defenses, RNA-seq, transcriptome profiles, neurotransmitter receptor

## Abstract

The crustacean *Daphnia pulex* is one of the best model organisms for studying inducible defense mechanisms due to their inducible morphology in response to the predator *Chaoborus* larvae. In this study, multiple developmental stages of *D. pulex* were exposed to *C. flavicans* larvae and transcriptome profiles of samples from late embryo to fifth instar were sequenced by the RNA-seq technique to investigate the genetic background underlying inducible defenses. In comparison, differentially expressed genes between defensive and normal morphs were identified, including 908 genes in late embryo, 1383 genes in the first-third (1–3) instar, and 1042 genes in fourth-fifth (4–5) instar. Gene ontology enrichment analysis showed that structural constituents of the cuticle and structural molecule activity genes were prominent up-regulated genes in late embryos. Down-regulated genes in late embryos and 1–3 instar comprised metabolic process, hydrolase activity, and peptidase activity gene classes. Pathway analysis indicated that small molecule neurotransmitter pathways were potentially involved in the development of inducible defenses. The characterization of genes and pathways in multiple developmental stages can improve our understanding of inducible defense responses of *D. pulex* to predation at the molecular level.

## 1. Introduction

The ability of expressing different phenotypes from the same genotype due to environmental stimulus is known as phenotypic plasticity, or polyphenism [1]. Much effort has been concentrated on mechanisms and consequences of phenotypic plasticity for better understanding of evolution and maintenance of biodiversity [2]. The water flea *Daphnia* is one of the most familiar model organisms for studying phenotypic plasticity [3]. They have many defense strategies against their predators, such as alterations in morphology, life-history, and behavior [4]. However, morphological alteration is considered as the most distinctive example of phenotypic plasticity in *Daphnia*. Several *Daphnia* species change their morphology in response to predator cues [5]. Of these species, *D. pulex* forms neckteeth on the back of the neck as a defense mechanism against predation when exposed to kairomones emitted by *Chaoborus* larvae [6,7].

Previous studies suggested that *Daphnia* is sensitive to kairomones in the embryonic stage and physiological changes occur through the activities of neuronal and endocrine factors [8,9,10]. The cholinergic pathway, GABA-ergic pathway, and glutamatergic pathway were reported to be involved in the perception and transmission of kairomones emitted by predators [9,11,12]. Additionally, the endocrine systems as juvenile hormones and ecdysone were also known to participate in regulating the formation of defensive morph [10,13]. However, the molecular genetics of inducible defense formation is poorly understood because the comprehensive genes and regulatory pathways have not been fully identified [14]. The availability of *D. pulex* genome information has fueled studies on the genetic mechanisms of defensive morph induction. In early studies, selected genes were investigated by real-time PCR to identify differentially expressed genes (DEGs) that mediate the morphological changes [8,15]. The development of next-generation sequencing technology has resulted in the usage of RNA-seq in unraveling large amounts of genes involved in the formation of defensive morph in *Daphnia* [16,17].

One of the common usages of RNA-seq technology is differential gene expression analysis [18]. Even though *D. pulex* has been studied in various fields, such as genetics, ecology, and toxicology [19,20,21,22], there is limited application of RNA-seq for the investigation of polyphenism induction. In a previous study, Rozenberg et al. [16] used RNA-seq to detect transcriptional changes in the first instar of *D. pulex* induced by *Chaoborus* kairomones. This study identified various genes that exhibited differential expression in defensive morph compared to normal morph. However, neckteeth development is a long process that spans multiple developmental stages. Late embryo is a critical stage for the induction of neckteeth formation with a kairomone-sensitive period [4,23]. The level of neckteeth formation is increased from the first instar to the third instar, then diminished in the fourth instar, and almost disappeared in the fifth instar [6,7,24]. Therefore, study of transcriptome profiles of multiple developmental stages might be helpful to understand the genetic background of inducible morphological defenses in *D. pulex*.

The aim of this study was to examine DEGs involved in the development of defensive morph of *D. pulex* in multiple developmental stages. For this aim, RNA-seq was performed to detect the transcriptome profiles of defensive and normal morphs from the late embryonic stage to the fifth instar juvenile. Genes and gene classes underlying inducible morphological defenses were investigated based on an analysis of transcriptome profiles. Moreover, neuronal pathways that potentially regulate the development of defensive morph were identified.

## 2. Results

### 2.1. RNA-Seq and Transcriptome Assembly

The RNA-seq data of the libraries; normal morph of late embryo (NML), normal morph of 1–3 instar (NM 1–3), normal morph of 4–5 instar (NM 4–5), defensive morph of late embryo (DML), defensive morph of 1–3 instar (DM 1–3), and defensive morph of 4–5 instar (DM 4–5) are presented in Table 1. In total, RNA-seq generated 327,210,512 raw reads for control and induced samples. After the trimming process, we obtained 42,012,059 reads of late embryo, 45,251,965 reads of 1–3 instar, 45,501,199 reads of 4–5 instar for normal morph samples, and 42,390,858 reads of late embryo, 41,815,412 reads of 1–3 instar, and 42,148,558 reads of 4–5 instar for defensive morph samples. From 259,120,051 clean reads, 141,448,402 (54.6%) reads were successfully mapped to the *D. pulex* reference genome (Table 2).

### 2.2. Relation of Gene Expression Profiles

Correlation analysis between libraries showed that gene expression profiles at the same developmental stage shared high similarity (Supplementary file: Figure S1). In particular, transcript profiles of late embryo, 1–3 instar, and 4–5 instar of normal morph were comparable to those of defensive morph. This finding is congruent to hierarchical clustering analysis that revealed the clustering of libraries at the same developmental stages (Supplementary file: Figure S2).

### 2.3. Differentially Expressed Genes (DEG) and Gene Ontology (GO) Enrichment Analysis

**Figure 1:**
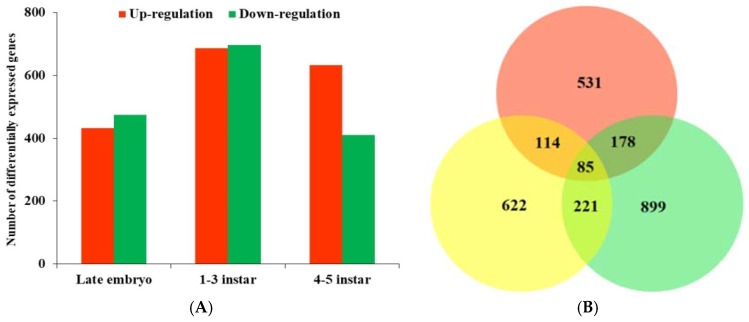
Number of DEGs (differentially expressed genes) in defensive morph of *D. pulex* by comparing to normal morph in each developmental stage (**A**). Diagram (**B**) shows the number of DEGs shared by late embryo (red), 1–3 instar (green), and 4–5 instar (yellow) stages.

GO enrichment analysis was conducted to retrieve the functional profiles of DEGs. Comparison between gene sets of defensive and normal morphs were performed to identify DEGs (fold-change > 2, *p* < 0.01) of *D. pulex* under predator stress (Supplementary file: Table S1). In total, 433 up-regulated and 475 down-regulated genes in late embryo, 687 up-regulated and 696 down-regulated genes in 1–3 instar, and 632 up-regulated and 410 down-regulated genes in 4–5 instar were found (Figure 1A). The three-set Venn diagram indicated that 85 genes were shared among all stages (Figure 1B). Moreover, the shared genes among stages showed bias, with the highest number between 1–3 instar and 4–5 instar.

**Figure 2:**
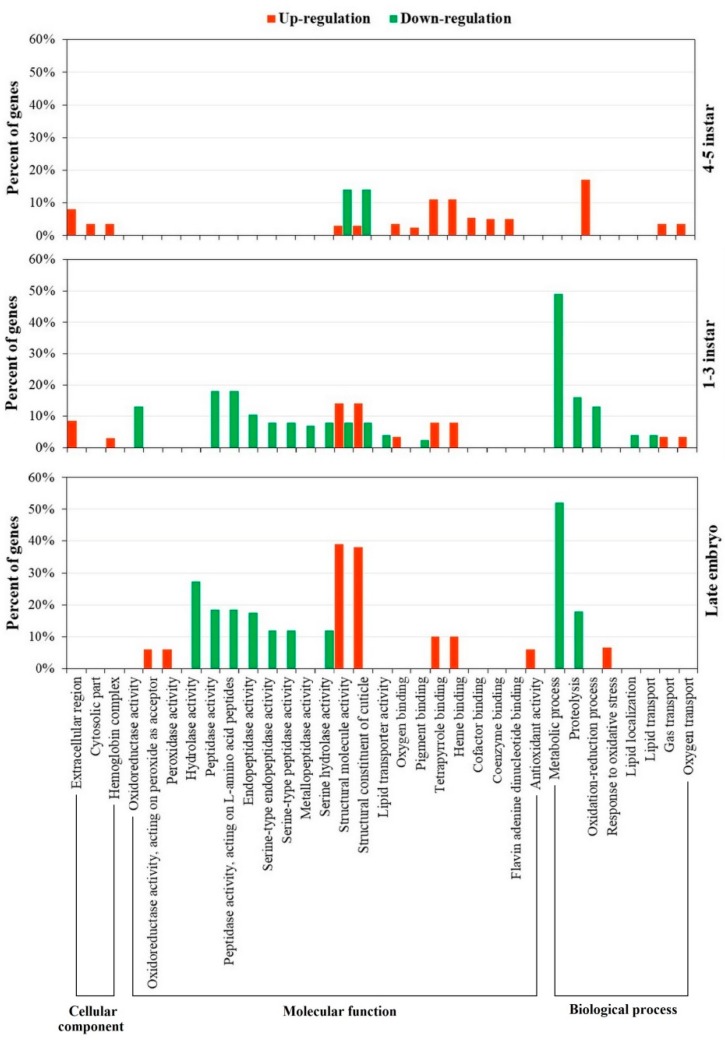
GO (Gene Ontology) enrichment analysis (FDR (false discovery rate) < 0.05) for the DEGs (fold change > 2, *p* < 0.01) in each developmental stage. Red and green represent up-regulated and down-regulated genes in defensive morph, respectively. The *x* axis shows enriched GO terms and *y* axis show the percentage of up- and/or down-regulated genes. The percentages of regulated genes were calculated individually for every GO term.

Significantly over-expressed gene classes in different developmental stages are presented in Figure 2. There are three main groups that include 33 GO categories: cellular component, molecular function, and biological process. In late embryos, nearly 40% of structural constituents of cuticle and structural molecule activity genes showed up-regulation. Genes with antioxidant functions, such as peroxidase activity and antioxidant activity, were also up-regulated in this stage. In contrast, metabolic process and hydrolase activity genes exhibited a high percentage of down-regulation. In particular, more than 50% and 20% of metabolic process and hydrolase activity genes were down-regulated, respectively. In 1–3 instar, structural constituents of cuticle and structural molecule activity genes showed both up-regulated and down-regulated patterns with more up-regulated genes. A high percentage of down-regulated genes were observed in metabolic processes, peptidase activity, and peptidase activity, acting on L-amino acid peptides. In the 4–5 instar stage, less than 20% of genes in each category exhibited differential expression. Most GO categories were up-regulated, except constituents of cuticle and structural molecule activity genes presented mixed expression patterns.

### 2.4. Neurotransmitter Receptors

There were 21 neurotransmitter receptor genes that exhibited differential expression in defensive morph compared to normal morph (Supplementary file: Table S1). The neurotransmitter receptor genes consisted of three sub-groups: peptide receptors, amine receptors, and small molecule receptors. Allatostatin receptors, ecdysis triggering hormone receptors, and myosuppressin receptors, belong to the peptide receptor sub-group, while glutamate receptors, such as kainate, *NMDA*, *AMPA*, metabotropic glutamate, belong to small molecule receptor sub-group.

### 2.5. Small Molecule Neurotransmitter Pathway Analysis

DEGs were mapped to the Kyoto Encyclopedia of Genes and Genomes (KEGG) database to detect neuronal pathways that regulate the development of defensive morph. DEGs and their regulation in glutamatergic pathway were presented in Table 3. A total of 18 DEGs were mapped to nine pathway genes with a majority of glutamate receptors (*AMPA*, *NMDA*, kainate, and metabotropic glutamate). Of genes coding for glutamate receptors, *AMPA* receptor gene was up-regulated in the 1–3 instar stage while the NMDA receptor genes were up-regulated in late embryo and 4–5 instar stages. On other hand, kainate receptor genes showed different regulation patterns among developmental stages. Genes coding for mitogen-activated protein kinase (*Erk*), inositol 1,4,5-triphosphate receptor (*IP3R*), RAS GTPase (RAS), nicotinic acetylcholine receptor α-8 precursor (*nAChR*), nicotinic acetylcholine receptor α-3 (*nAChR*), and protein kinase A (PKA) were involved in the cholinergic pathway (Table 4). Of *nAChR* genes, nicotinic acetylcholine receptor α-8 precursor was up-regulated in the 1–3 instar stage, whereas nicotinic acetylcholine receptor α-3 was down-regulated in the 4–5 instar stage. In the GABA-ergic pathway, GABA transaminase (GABA-T), protein kinase A, and adenylate cyclase genes were reported (Table 5). GABA transaminase genes showed different regulation patterns in late embryo and up-regulation in 1–3 instar and 4–5 instar stages.

### 2.6. Real-Time PCR Validation of RNA-Seq Data

Real-time PCR was conducted to validate the significance of RNA-seq data. Twelve genes were randomly selected to run real-time PCR (Supplementary file: Table S2). The gene expression patterns of Real-time PCR were consistent with those of RNA-seq (Supplementary file: Figure S3).

## 3. Discussion

In RNA-seq, RNA information of biological samples is generated from cDNA sequences using high-throughput sequencing technologies [25,26]. As an advanced technique, RNA-seq has been widely applied to study genetic responses of organisms to environmental variations. The current study used RNA-seq to investigate the molecular basis of predator-induced polyphenism from the late embryo to fifth instar stage of *D. pulex*. The abundance and significant over-representation of cuticle-encoded genes were found among developmental stages (Figure 2). Rozenberg et al. [16] discovered that cuticle-associated transcripts were the most abundant group of up-regulated genes in *D. pulex* confronted with *Chaoborus* larvae. The current study showed the prevalence of genes coding for structural constituent of cuticle and structural molecule activity among up-regulated genes in late embryo. The up-regulation of structural genes in the late embryo stage can be explained by neckteeth formation. It is known that the development of neckteeth is triggered during embryogenesis [5,9]. When late embryo *D. pulex* is exposed to kairomones, the epidermal cells of the neckteeth-forming area proliferate and form neckteeth [4,5]. Therefore, the expressions of structural genes are enhanced to meet the increased demand of structural molecules for neckteeth formation.

In late embryo, the up-regulation of antioxidant-related genes, such as peroxidase activity and antioxidant activity genes, were also observed (Figure 2). Under predator risk, an increase in oxygen consumption of prey may result in oxidative stress [27]. The over-representation of the response to oxidative stress genes was an indicator for oxidative stress occurring in *D. pulex* (Figure 2). The increased expression of antioxidant-related genes may help the water flea reduce detrimental effects of oxidative stress caused by *C. flavicans*. It is reported that antioxidant-related genes were increased after *Daphnia* was exposed to environmental stressors [17,28,29].

The down-regulation of metabolism-associated genes in the late embryo and 1–3 instar stages indicated the reduction of metabolism in these stages (Figure 2). Due to the cost of neckteeth formation and maintenance [3,30], metabolic processes in *D. pulex* may be affected. Preys under predation stress have tendency to reduction in rates of growth or development [31]. Previous study demonstrated that the population growth rate in *Daphnia* was hampered by fish kairomones [32]. Moreover, the changes of hydrolase activities indicated that the metabolism was interfered by environmental stressors [33,34,35]. The down-regulation of hydrolase activity expression genes observed in this study revealed the effects of *C. flavicans* kairomones on metabolic processes in *D. pulex*.

There were 21 neurotransmitter receptor genes that showed differential expression between defensive and normal morphs (Supplementary file: Table S1). Neuronal signal transmission is proven to link with kairomone reception and neckteeth formation [11,12,13]. Genes for allatostatin receptors and ecdysis triggering hormone receptors were identified among neurotransmitter receptor genes. Ecdysis triggering hormone is known to regulate ecdysis in arthropods [36,37] whereas the function of allatostatin hormone is to inhibit juvenile hormone synthesis [38]. Juvenile hormone is a major hormone that regulates the development and morphogenesis in insects and crustaceans [39,40,41] and promotes neckteeth formation in *D. pulex* [13]. Additionally, we also discovered the differential expressions of genes coding for *AMPA*, *NMDA*, kainate, and metabotropic glutamate receptors between defensive and normal morphs. *AMPA*, *NMDA*, kainate, and metabotropic glutamate are members of glutamate receptors that perform the function at the central synapses as mediation factors of fast excitatory transmission [42,43].

Neurotransmitter receptors are activated by binding to neurotransmitters. Small molecule neurotransmitters, such as acetylcholine, glutamate, and GABA, were suggested to involve in neckteeth formation [44,45]. The different regulations of glutamatergic pathway genes exhibited the potential involvement of this pathway in the inducible defenses of *D. pulex* (Table 3). Glutamate receptors are divided into metabotropic and ionotropic glutamate receptors, in which ionotropic glutamate receptors comprise *AMPA*, NMDA, and kainate receptors [46]. Of glutamate receptor genes, there was up-regulation of *AMPA* in 1–3 instar and NMDA in late embryo and 4–5 instar, while kainate presented differential expression patterns among developmental stages (Table 3). Chiang et al. [47,48] revealed that the cockroach *Diploptera punctata* produced a high amount of juvenile hormone in response to NMDA and kainate, leading to the conclusion that NMDA and kainate receptors regulate juvenile hormone synthesis. In *D. pulex*, ionotropic glutamate receptors were reported to mediate the formation of defensive morph [9].

In addition to glutamatergic pathway, cholinergic pathway was shown to potentially engage in neckteeth formation. In this pathway, nicotinic acetylcholine receptors were subject to up-regulation in 1–3 instar and down-regulation in 4–5 instar stages, corresponding to the appearance and disappearance of neckteeth (Table 4). Previous reports demonstrated that cholinergic pathway participates in the detection and transmission of kairomones [11,12]. Accordingly, neckteeth development was boosted by the stimulation of cholinergic transmission and suppressed by the inhibition of cholinergic transmission.

In the GABA-ergic pathway, the differential expression of GABA transaminase genes was found in all developmental stages (Table 5). GABA transaminase is an enzyme responsible for catabolism of γ-aminobutyric acid (GABA), a main inhibitory neurotransmitter in the central nervous system [49]. The involvement of GABA in neckteeth formation is still controversial since different studies indicate dissimilar results [9,11,12]. Weiss et al. [12] showed that GABA did not participate in the perception and transmission of *Chaoborus* kairomones. In contrast, Miyakawa et al. [9] and Barry [11] expressed the important roles of GABA in the formation of defensive morph. Our findings suggest that GABA-ergic elements have a relationship with predator-induced polyphenism of *D. pulex*, although it is not as clear as the involvements of glutamatergic and cholinergic elements.

Multiple neuronal factors found in this study are potentially involved in regulating the development of defensive morph of *D. pulex*. The formation of inducible defenses requires various components for kairomone perception and activation of defensive mechanisms [8,45]. Weiss et al. [45] proposed a pathway starting with the perception of predator cues, followed by neuronal signal alterations in the central nervous system and, finally, neuro-hormonal changes. Therefore, the phenotypic outcome in *D. pulex* is suggested to mirror the neuro-hormonal responses to predator cues [45]. Further studies should concentrate on unraveling the role of hormonal factors in the formation and growth of defensive morph.

## 4. Materials and Methods

### 4.1. Ethical Statement

*Daphnia pulex* was provided by Daphnia Stock Center (Daphnia Genomics Consortium), University of South Carolina, Colombia, USA. *D. pulex* and *C. flavicans* are not endangered or protected species. The use of *D. pulex* and *C. flavicans* did not require approval from an ethics committee.

### 4.2. Daphnia pulex and Chaoborus flavicans Culture

The *D. pulex* TRO (The Rejected One) clone used in this study was obtained from Daphnia Stock Center (Daphnia Genomics Consortium), University of South Carolina, Columbia, SC, USA. *D. pulex* was cultured in M4 medium at 20 °C and 16 h/8 h light/dark cycle [50]. Each day, the cultured organism was fed with 100 mL of the green algae *Scenedesmus* sp. provided by Korean Marine Microalgae Culture Center, Busan, Korea.

*C. flavicans* larvae were collected from the Sangcheon reservoir (37°46′05′′ N, 127°29′32′′ E) in Gapyeong and the Ildae reservoir (35°31′33′′ N, 127°35′47′′ E) in Namwon, Korea. The phantom midge larvae were cultured as *D. pulex*, and fed with approximately 100 *D. pulex* larvae daily.

### 4.3. Induction of Defensive Morph

Embryo-bearing *D. pulex* were placed in a 3 L beaker and a nylon cage (mesh size 200 μm) containing 40 fourth instar phantom midge larvae, put in the same beaker to induce the formation of neckteeth in *D. pulex* [51,52]. The cage prevented *C. flavicans* larvae prey on *D. pulex* and *D. pulex* were exposed to the kairomones through the cage. Only first instar individuals with neckteeth obtained from the induced parents were transferred to a new beaker and grown in the same condition with the availability of *C. flavicans* larvae. This procedure was repeated until *D. pulex* reached the fifth instar stage. The control group was cultured under the same conditions as the experimental group without *C. flavicans* in the cage.

### 4.4. RNA-Seq Library Preparation and Illumina Sequencing

After the culturing process, approximately 200 late embryo individuals, 130 first instar individuals, 100 second instar individuals, 60 third instar individuals, 40 fourth instar individuals, and 30 fifth instar individuals of both defensive and normal morphs were collected for transcriptomic analysis. Total RNA was extracted from pooled samples at each developmental stage for normal and defensive morph using TRIzol reagent (Invitrogen, Carlsbad, CA, USA) following the manufacturer’s instruction. RNA quality was determined by the RNA integrity number (RIN) of a 2100 Bioanalyzer system (Agilent Technologies, Santa Clara, CA, USA) with a minimum integrity number value of 6. Illumina’s TruSeq RNA Sample Preparation kit v2 was used to prepare cDNA libraries according to manufacturer’s protocol. Since neckteeth of *D. pulex* are formed and increased from 1–3 instar and decreased from 4–5 instar, 1–3 instar libraries were pooled and 4–5 instar libraries were pooled. Subsequently, six cDNA libraries were targeted for paired-end sequencing by Illumina HiSeq 2000 (Illumina Inc., San Diego, CA, USA). All raw data obtained by RNA-seq was deposited in NCBI Sequence Read Archive, accession number: PRJNA241046.

### 4.5. Raw Data Processing and Functional Annotation

The low-quality or contaminated reads retrieved from RNA-seq were removed by a two-step trimming process. The first step was performed to remove adaptor sequences and low-quality reads: unknown nucleotides (N) accounting for more than 5% of bases, reads less than 80 bases in length, and reads with more than 10% of bases below Q20 quality. In the second step, to remove contaminated reads, the previously trimmed reads were mapped to Algae DB (JGI Heterokont, JGI Chlorella, JGI Chloropyta, and JGI Viridiplantae) of the JGI database and the bacteria, virus, and fungi DB of the NCBI database. Based on the mapping results, reads with more than 90% identity were discarded.

The genome of *D. pulex* published by the Department of Energy Joint Genome Institute (JGI) [52] was used for reference-guided assembly. Mapping and assembly were performed with TopHat 1.3.2 [53] and Cufflinks 1.3.0 [54]. The functions of *D. pulex* genes were detected with the JGI V11 model of the Daphnia genome database (wFleaBase) [55]. For genes with unknown functions in wFleaBase, Pedant-Pro was used to predict their functions [56].

### 4.6. Comparison of Gene Expression Profiles

The expression level of each gene was measured by the reads per kilobase per million mapped reads method (RPKM) [57]. Pearson correlation analysis and hierarchical clustering analysis was performed with GeneSpring 12 (Agilent Technologies, Santa Clara, CA, USA) to investigate the relation of gene expression among six libraries.

### 4.7. Identification of DEGs

The analysis of DEGs aids to elucidate genes involved in inducible defenses. To identify DEGs, the fold change of genes was calculated by comparing gene’s RPKM values between defensive and normal morphs for each developmental stage. Audic’s method was applied to evaluate the statistical significance [58]. Genes with a fold change higher than 2 and a *p*-value lower than 0.01 were selected as DEGs.

### 4.8. Gene Ontology Enrichment Analysis

GO enrichment analysis was conducted using the BLAST2GO program to unravel the statistically significant enrichment of DEGs [59]. The whole *D. pulex* transcriptome was set as a reference while DEGs of each library were set as a target for Fisher’s exact test with a false discovery rate (FDR) cutoff of 0.05.

### 4.9. Pathway Analysis

DEGs were used to search for neuronal pathways involved in the development of defensive morph. Pathway analysis was performed with the KEGG database. We also identified the regulation of DEGs among developmental stages for each pathway.

### 4.10. Validation of DEGs Using Real-Time PCR

Real-time PCR was performed with twelve genes, which were randomly selected from the list of DEGs, to confirm the significance of RNA-seq results. The primers and descriptions for each gene are presented in the Supplementary file, Table S2. Glyceraldehyde-3-phosphate dehydrogenase (GAPDH; AJ289783) was used as a reference gene [8]. A reaction mixture of 20 μL included 10 μL of 2X SYBR Green PCR Master Mix (PE Applied Biosystems, Waltham, MA, USA), 0.8 μL of 10 pmol/μL of primers, 0.5 μL of cDNA, and 8.7 μL of distilled water. Real-time PCR was conducted using a 7900HT Sequence Detection System (PE Applied Biosystems, Waltham, MA, USA) with triplicates of each reaction. The PCR conditions consisted of a 5 min. denaturation step at 95 °C, followed by 40 cycles of 95 °C for 30 seconds, 55 °C for 30 s, and 72 °C for 30 s, and a final extension at 72 °C for 5 min. Relative expression of transcripts was calculated using the 2^–∆∆*C*t^ method through quantification cycle (Cq) values [60]. The significance of differences was analyzed with a pairwise *t*-test (*p* < 0.05) using the PASW Statistics 18 program (SPSS Inc., Chicago, IL, USA).

## 5. Conclusions

RNA-seq was applied to investigate the genetic information underlying predator-induced polyphenism in the multiple developmental stages of *D. pulex*. In particular, we discovered different genes and gene classes involved in inducible defenses from late embryo to fifth instar stages. Further, neuronal pathways that potentially regulate the formation of defensive morph were uncovered. Data obtained in this study increases our understanding of gene expression occurring in *D. pulex* under predator risk. Additionally, the results provide a fundamental basis for studies on the genetic background of predator-induced polyphenism in various organisms in the future.

## Figures and Tables

**Table 1:** The statistics of raw data and trimmed data.

Libraries	Paired-End Reads Before Trimming	Trimmed Paired-End Reads	Trimmed Single-End Reads	Total Trimmed Reads
NML	49,868,688	38,531,810 (77.3%)	3,480,249 (7.0%)	42,012,059 (84.2%)
NM1–3	53,366,508	41,745,502 (78.2%)	3,506,463 (6.6%)	45,251,965 (84.8%)
NM4–5	53,291,167	42,142,048 (79.1%)	3,359,151 (6.3%)	45,501,199 (85.4%)
DML	57,457,116	37,663,210 (65.6%)	4,727,648 (8.2%)	42,390,858 (73.8%)
DM1–3	56,210,099	37,198,644 (66.2%)	4,616,768 (8.2%)	41,815,412 (74.4%)
DM4–5	57,016,934	37,459,438 (65.7%)	4,689,120 (8.2%)	42,148,558 (73.9%)
Total	327,210,512	234,740,652 (71.7%)	24,379,399 (7.5%)	259,120,051 (79.2%)

NML (normal morph of late embryo), NM1–3 (normal morph of 1–3 instar), NM4–5 (normal morph of 4–5 instar), DML (defensive morph of late embryo), DM1–3 (defensive morph of 1–3 instar), and DM4–5 (defensive morph of 4–5 instar).

**Table 2:** Results of mapping reads to the *D. pulex* genome.

Libraries	Mapped Reads	Mapped Genes
NML	23,508,160 (56.0%)	16,139 (52.2%)
NM 1–3	24,761,033 (54.7%)	16,153 (52.3%)
NM 4–5	24,143,663 (53.1%)	16,400 (53.1%)
DML	25,006,291 (59.0%)	16,112 (52.1%)
DM 1–3	22,349,587 (53.4%)	16,284 (52.7%)
DM 4–5	21,679,668 (51.4%)	16,427 (53.1%)
Total	141,448,402 (54.6%)	18,207 (58.9%)

NML (normal morph of late embryo), NM 1–3 (normal morph of 1–3 instar), NM 4–5 (normal morph of 4–5 instar), DML (defensive morph of late embryo), DM 1–3 (defensive morph of 1–3 instar), and DM 4–5 (defensive morph of 4–5 instar).

**Table 3:** DEGs in glutamatergic pathway.

Gene ID	Late Embryo	1–3 Instar	4–5 Instar	Pathway Genes	Descriptions
e_gw1.374.3.1	Up	-	-	*NMDAR*	NMDA receptor subunit GluN2 *
NCBI_GNO_10400049	Up	-	-	*IP3R*	Inositol 1.4.5- trisphosphate receptor *
estExt_fgenesh1_pm.C_140020	Up	-	-	*Erk*	Mitogen-activated protein kinase **
NCBI_GNO_8900043	Down	-	-	*KA*	Kainate receptor subunit GluR8 *
NCBI_GNO_3900088	-	Up	-	*AMPA*	AMPA receptor subunit GluR2 *
NCBI_GNO_18700035	-	Up	-	*KA*	Kainate receptor subunit GluR10 *
fgenesh1_pg.C_scaffold_183000025	-	Down	-	*KA*	Kainate receptor subunit GluR5 *
NCBI_GNO_2300006	-	Down	-	*KA*	Kainate receptor subunit GluR7 *
PASA_GEN_1700130	-	Down	-	*mGluR2/3*	Metabotropic glutamate receptor subunit GRM2 *
fgenesh1_pg.C_scaffold_92000012	-	Down	-	*SHANK*	SH3 and multiple ankyrin repeat domains protein *
fgenesh1_pg.C_scaffold_39000059	-	Down	-	*AC*	Adenylate cyclase **
estExt_fgenesh1_pg.C_1830028	-	Down	-	*PKA*	Protein kinase A *
NCBI_GNO_8300040	-	-	Up	*NMDAR*	NMDA receptor subunit GRIN1 *
estExt_fgenesh1_pg.C_1130025	-	-	Up	*NMDAR*	NMDA receptor subunit GluN2 *
NCBI_GNO_8300009	-	-	Up	*KA*	Kainate receptor subunit GluR5 *
NCBI_GNO_1700146	-	-	Up	*KA*	Kainate receptor subunit GluR6 *
NCBI_GNO_0900239	-	-	Up	*KA*	Kainate receptor subunit GluR9 *
NCBI_GNO_1500242	-	-	Down	*KA*	Kainate receptor subunit GluR5 *

Desciptions of DEGs are referred from wFleaBase (*), uniprot (**).

**Table 4:** DEGs in cholinergic pathway.

Gene ID	Late Embryo	1–3 Instar	4–5 Instar	Pathway Genes	Descriptions
estExt_fgenesh1_pm.C_140020	Up	-	-	*Erk*	Mitogen-activated protein kinase **
NCBI_GNO_10400049	Up	-	-	*IP3R*	Inositol 1.4.5-trisphosphate receptor *
estExt_fgenesh1_pg.C_70190	Down	Up	-	*RAS*	RAS GTPase *
fgenesh1_pg.C_scaffold_62000026	-	Up	-	*nAChR*	Nicotinic acetylcholine receptor α-8 precursor **
NCBI_GNO_2400096	-	Up	-	*RAS*	RAS GTPase *
estExt_fgenesh1_pg.C_1830028	-	Down	-	*PKA*	Protein kinase A *
e_gw1.76.84.1	-	-	Down	*nAChR*	Nicotinic acetylcholine receptor α-3 **

Descriptions of DEGs are referred from wFleaBase (*), uniprot (**).

**Table 5:** DEGs in GABA-ergic pathway.

Gene ID	Late Embryo	1–3 Instar	4–5 Instar	Pathway Genes	Descriptions
fgenesh1_pm.C_scaffold_46000013	Down	-	Up	*GABA-T*	GABA transaminase *
gw1.100.93.1	Up	-	-	*GABA-T*	GABA transaminase *
estExt_Genewise1.C_360108	-	Up	Up	*GABA-T*	GABA transaminase *
estExt_fgenesh1_pg.C_1830028	-	Down	-	*PKA*	Protein kinase A *
fgenesh1_pg.C_scaffold_39000059	-	Down	-	*AC*	Adenylate cyclase **

Descriptions of DEGs are referred from wFleaBase (*), uniprot (**).

## Supplementary Materials

Supplementary materials can be found at http://www.mdpi.com/1422-0067/19/7/2110/s1.

## Abbreviations

DEGs	Differentially expressed genes
1–3 instar	First to third instar
4–5 instar	Fourth to fifth instar
RPKM	Reads per kilobase per million mapped reads
GO	Gene ontology
KEGG	Kyoto Encyclopedia of Genes and Genomes
GABA	γ-Aminobutyric acid
