# Supplementary material for: Comparative Transcriptome Analysis for Understanding Predator-Induced Polyphenism in the Water Flea Daphnia pulex
Source: Int J Mol Sci. 2018 Jul 20;19(7):2110. doi: 10.3390/ijms19072110 (PMC6073494; doi:10.3390/ijms19072110)
Supplement: Supplementary file 1 [file ijms-19-02110-s001.zip › ijms-325902-supplementary-proofread.pdf]

Supplementary Materials

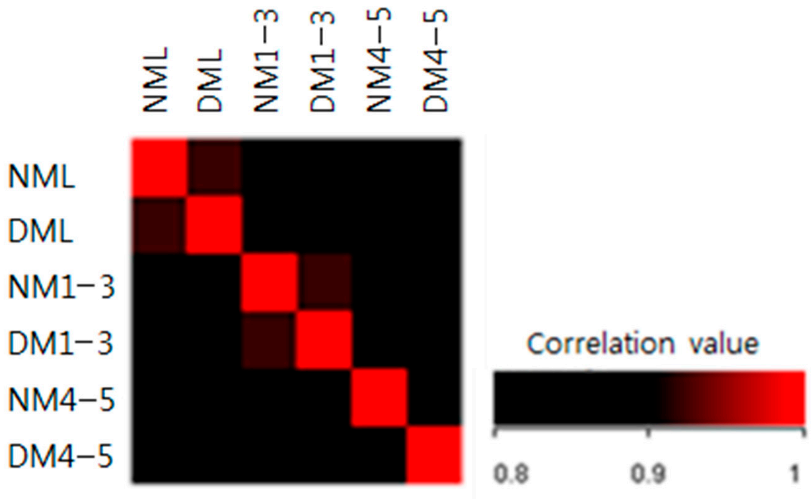

Figure S1. Pairwise correlation of the six libraries; NML (normal morph of late embryo), DML (defensive morph of late embryo), NM1-3 (normal morph of 1–3 instar), DM1-3 (defensive morph of 1–3 instar), NM4-5 (normal morph of 4–5 instar), and DM4-5 (defensive morph of 4–5 instar). Color changes from black to red mean correlation values from low to high.

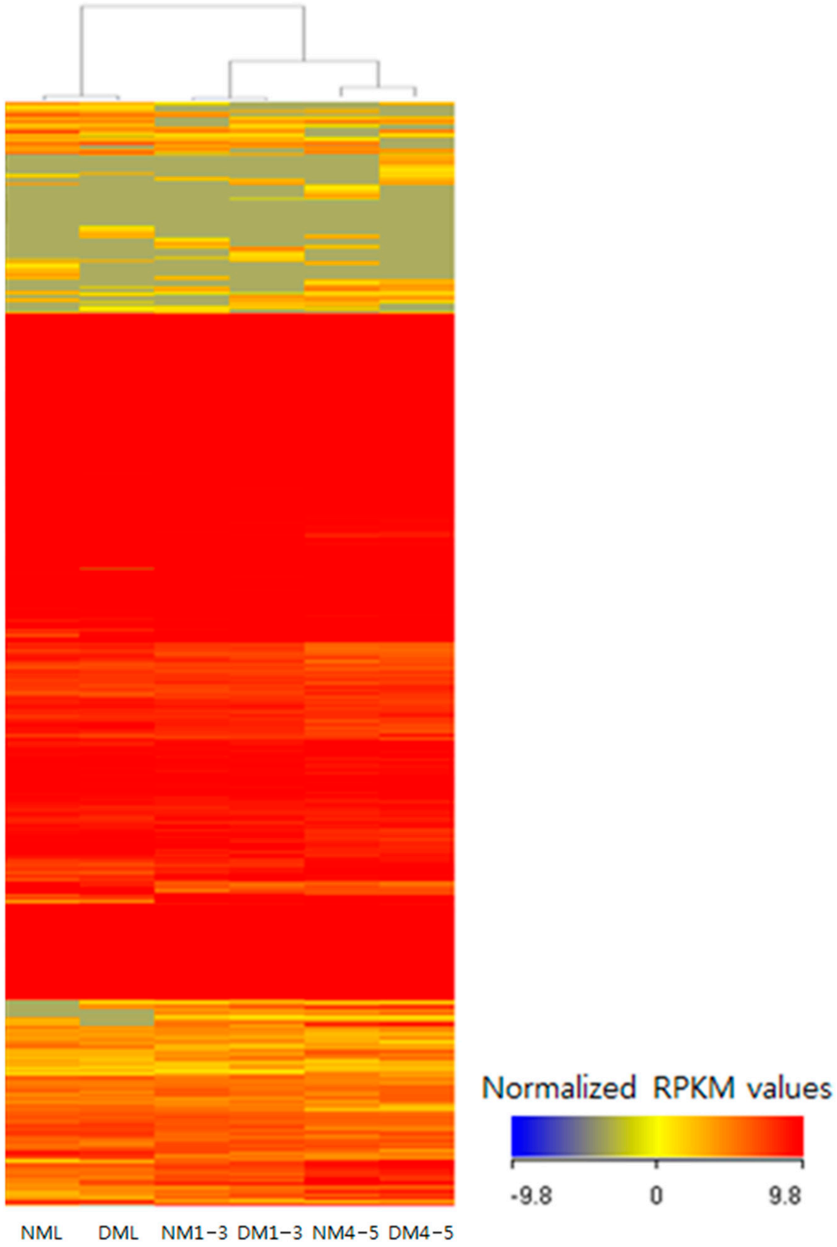

Figure S2. Hierarchical cluster of the expressed genes in the six libraries; NML (normal morph of late embryo), DML (defensive morph of late embryo), NM1-3 (normal morph of 1–3 instar), DM1-3 (defensive morph of 1–3 instar), NM4-5 (normal morph of 4–5 instar), and DM4-5 (defensive morph of 4-–5 instar). Color changes from blue to red represent normalized RPKM values from low to high.

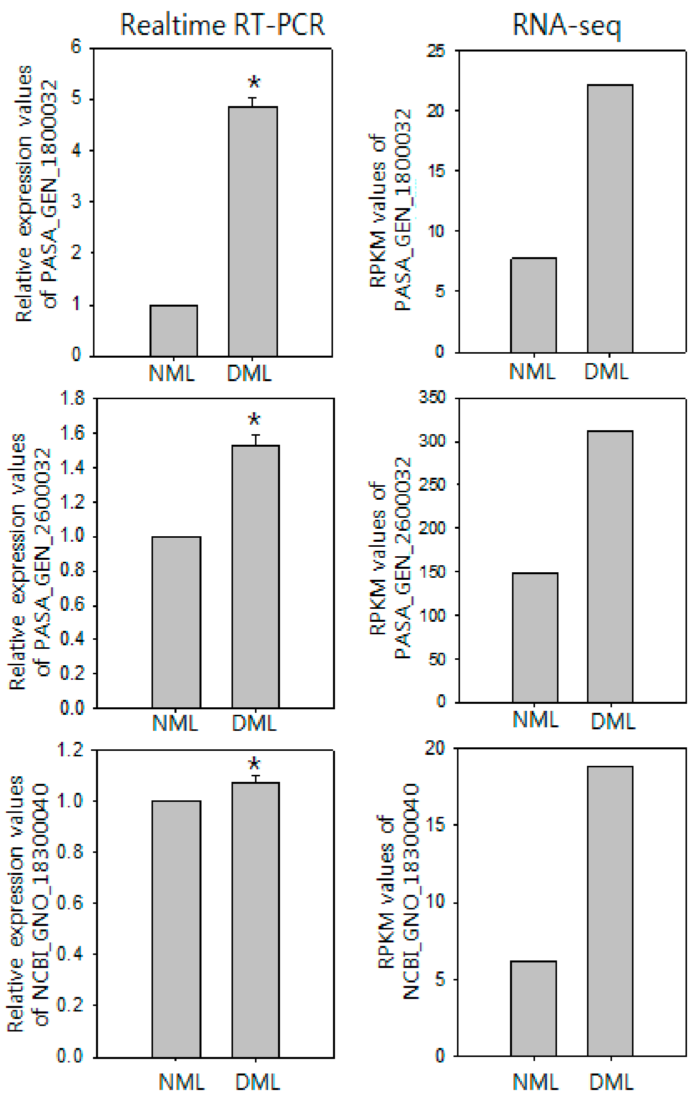

Figure S3. Real-time RT-PCR validation. * represents differentially-expressed genes between normal morph (NM) and defensive morph (DM) with significance (p < 0.05). NML and DML represent the normal morph of the late embryo and the defensive morph of the late embryo, respectively.

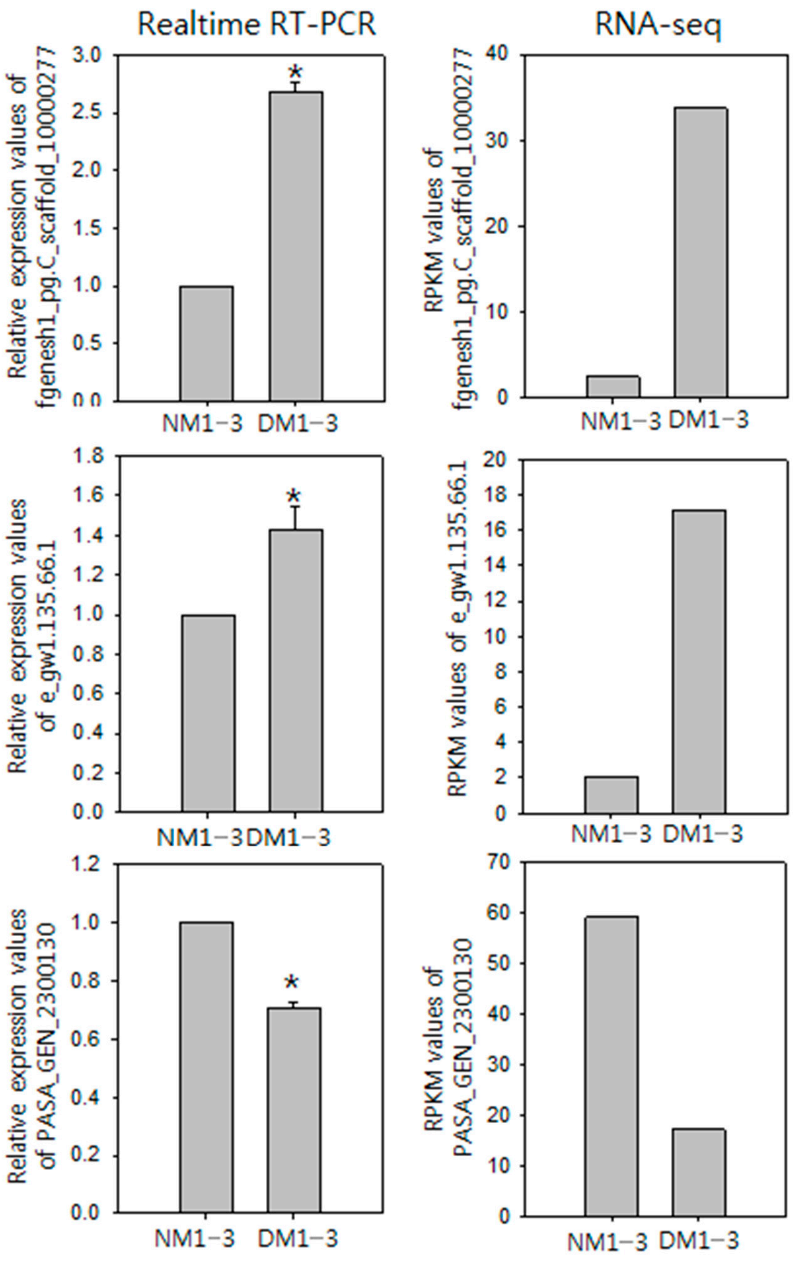

Figure S3. Real-time PCR validation. * represents differentially-expressed genes between normal morph (NM) and defensive morph (DM) with significance (p < 0.05). NM1-3 and DM1-3 represent the normal morph of 1–3 instar and the defensive morph of 1–3 instar, respectively.

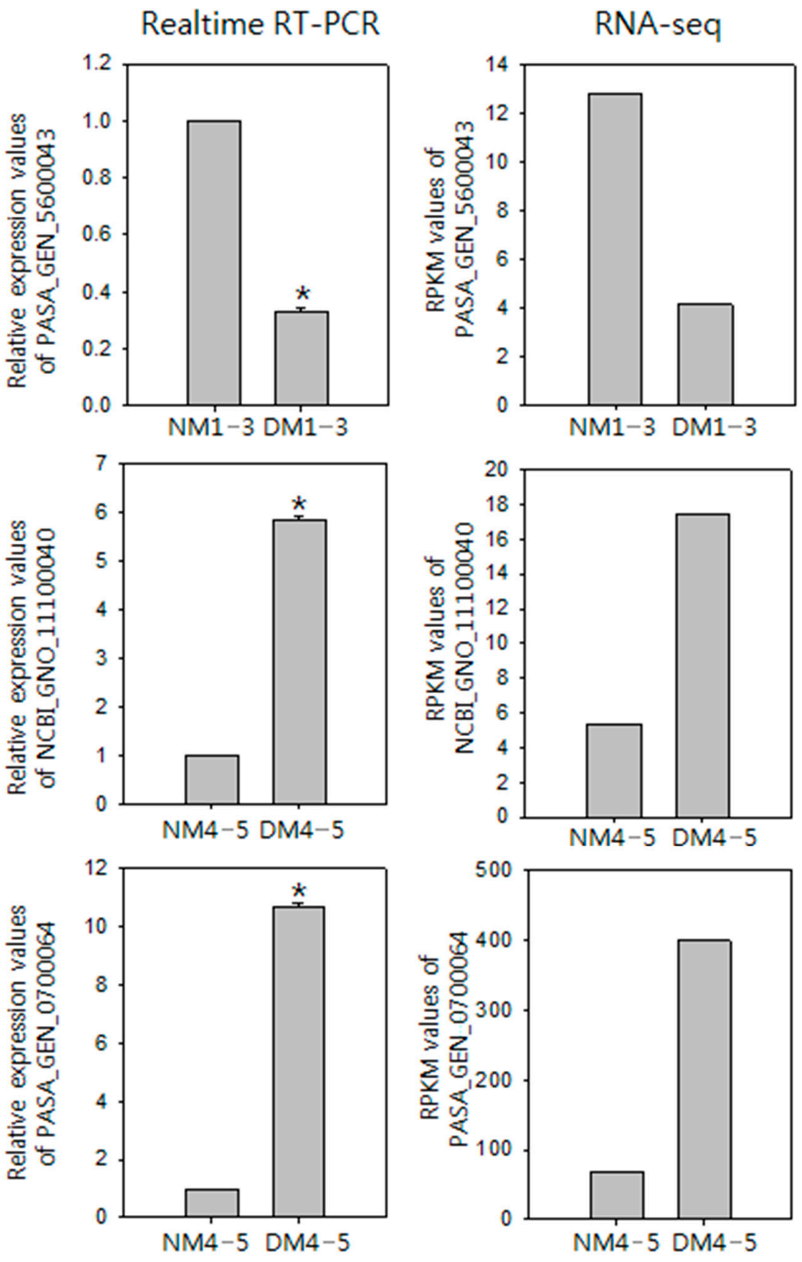

Figure S3. Real-time PCR validation. * represents differentially-expressed genes between the normal morph (NM) and the defensive morph (DM) with significance ($p < 0.05$). NM1-3, DM1-3, NM4-5, and DM4-5 represent the normal morph of 1–3 instar, defensive morph of 1–3 instar, normal morph of 4–5 instar, and defensive morph of 4–5 instar, respectively.

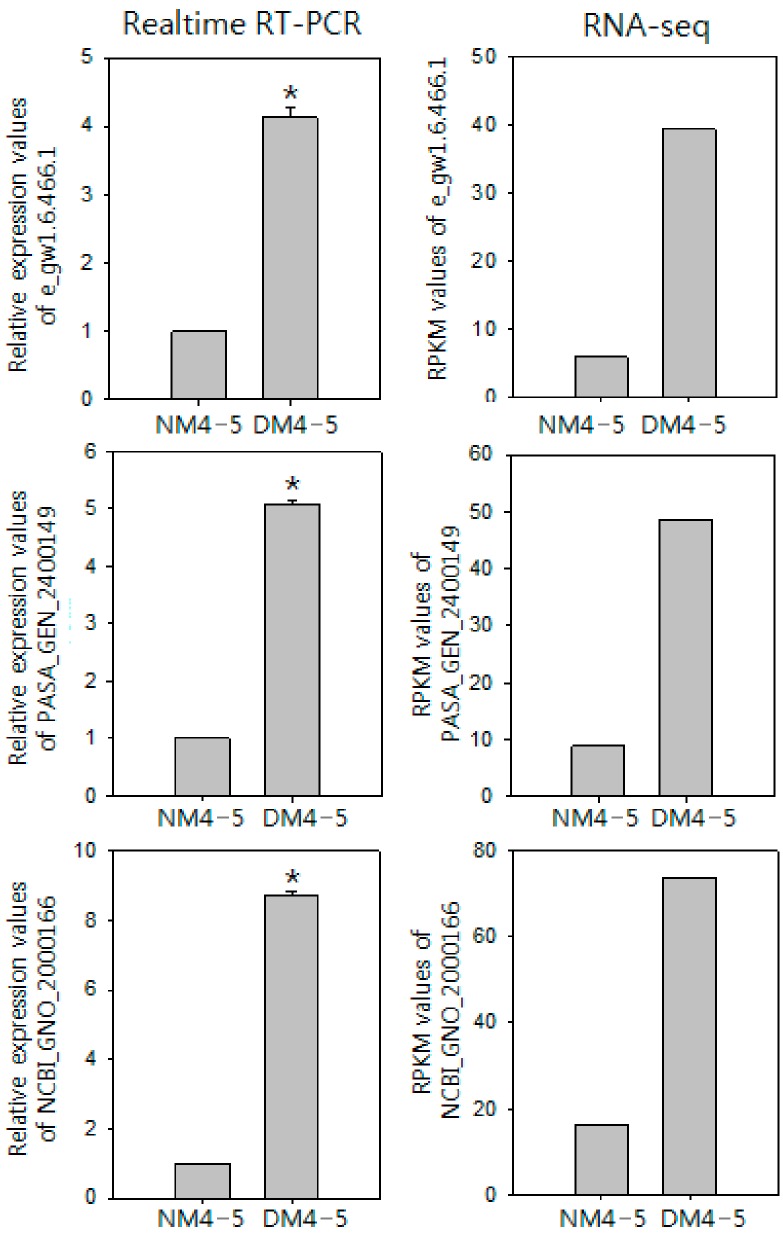

Figure S3. Real-time PCR validation. * represents differentially-expressed genes between normal morph (NM) and defensive morph (DM) with significance ($p < 0.05$). NM4-5 and DM4-5 represent the normal morph of 4–5 instar, and defensive morph of 4–5 instar, respectively.

Table S1. The List of the differently-expressed genes.

See Table S1.

Table S2. Differential expression of neurotransmitter receptors between normal and defensive morphs among developmental stages.

	Gene ID	Late embryo	1-3 instar	4-5 instar	Descriptions
Peptide receptor	e_gw1.91.62.1	Down	-	-	Allatostatin receptor – *Periplaneta americana***
	fgenesh1_pg.C_scaffold_1416000002	-	-	Up	Allatostatin receptor – *Periplaneta americana***
	gw1.142.6.1	-	Down	-	Ecdysis triggering hormone receptor subtype B - *Aedes aegypti***
	gw1.8.84.1	-	-	Up	AGAP005229-PA (Myosuppressin receptor) - *Anopheles gambiae***
Amine receptor	e_gw1.93.6.1	-	Down	-	Histamine-gated chloride channel subunit - *Culex quinquefasciatus***
	e_gw1.4.443.1	Up	-	-	Octopamine receptor - *Apis mellifera***
Small molecule receptor	fgenesh1_pg.C_scaffold_62000026	-	Up	-	Nicotinic acetylcholine receptor subunit alpha8 precursor - *Gallus gallus***
	e_gw1.76.84.1	-	-	Down	Nicotinic acetylcholine receptor subunit alpha3 - *Bombyx mori***
	NCBI_GNO_1500242	-	-	Down	Kainate receptor subunit GluR5 and related subunits*
	fgenesh1_pg.C_scaffold_183000025	-	Down	-	Kainate receptor subunit GluR5 and related subunits*
	NCBI_GNO_8300009	-	-	Up	Kainate receptor subunit GluR5 and related subunits*
	NCBI_GNO_1700146	-	-	Up	Kainate receptor subunit GluR6 and related subunits*

NCBI_GNO_2300006	-	Down	-	Kainate receptor subunit GluR7 and related subunits*
NCBI_GNO_8900043	Down	-	-	Kainate receptor subunit GluR8 and related subunits*
NCBI_GNO_0900239	-	-	Up	Kainate receptor subunit GluR9 and related subunits*
NCBI_GNO_18700035	-	Up	-	Kainate receptor subunit GluR10 and related subunits*
NCBI_GNO_8300040	-	-	Up	NMDA receptor subunit GluN1 and related subunits *
e_gw1.374.3.1	Up	-	-	NMDA receptor subunit GluN2 and related subunits *
estExt_fgenesh1_pg.C_1 130025	-	-	Up	NMDA receptor subunit GluN2 and related subunits *
NCBI_GNO_3900088	-	Up	-	AMPA receptor subunit GluR2 and related subunits *
PASA_GEN_1700130	-	Down	-	Metabotropic glutamate receptor subunit GRM2 and related subunits*

Descriptions of DEGs are referred from wFleaBase (*), uniprot (**).

Table S3. List of primers used for real-time PCR.

Primers	Sequence (5′-3′)	Descriptions
GAPDH_F	GGTGCTGCCCAGAACATTAT	Glyceraldehyde-3-phosphate dehydrogenase
GAPDH_R	GACAGCCTTGATTTCGTCGT	
PASA_GEN_1800032_F	GAGCCATAGACGAGGTCTGC	Insulin-like***
PASA_GEN_1800032_R	CAAGGACGACGACTGATTGA	
PASA_GEN_2600032_F	GTGGTGAAGACATGGACGTG	Molecular chaperone (DnaJ superfamily)*
PASA_GEN_2600032_R	CAATGGGCGGATCTTGTACT	

NCBI_GNO_18300040_F	GGTCTCCTCCTCATGTCCAA	Predicted lipoprotein*
NCBI_GNO_18300040_R	GACTGGATCAGCTTGTGGGT	
fgenesh1_pg.C_scaffold_10000277_F	CAGCTCAGTTCAGACAAGCG	Calcium-responsive transcription coactivator*
fgenesh1_pg.C_scaffold_10000277_R	GGTCCTGCCATCTGATTCAT	
e_gw1.135.66.1_F	CTCCGTTTGCCGTTATGTTT	Na+/dicarboxylate. Na+/tricarboxylate and phosphate
e_gw1.135.66.1_R	GCTGCGTTTGAGGCTATTTC	transporters*
PASA_GEN_2300130_F	AATGCTCGCGTTAATTGGAC	Permease of the major facilitator superfamily*
PASA_GEN_2300130_R	CAGCGGTCATTGCAGTTAGA	
PASA_GEN_5600043_F	CTGGCATTTCCTTTGGATGT	Transcription factor BLIMP-1/PRDI-BF1. contains
PASA_GEN_5600043_R	ACCAGAATGGTGAGAAACGG	C2H2- type Zn-finger and SET domains*
NCBI_GNO_11100040_F	GCTCAAGGCTGTGGAAGAAG	Monocarboxylate transporter*
NCBI_GNO_11100040_R	AACCAAATGGACGTATGGGA	
sPASA_GEN_0700064_F	AGAATCCCACGTCCAGTGTC	Peroxidase*
PASA_GEN_0700064_R	CGTTGGTGAGTAGCACGAGA	
e_gw1.6.466.1_F	CGGCTGTGAAATCAGTCTCA	Ankyrin repeat*
e_gw1.6.466.1_R	TGAGGATTTCTGCAGGCTTT	
PASA_GEN_2400149_F	ATTGTCGATGAGCACAGCAG	Low-density lipoprotein receptors containing Ca2+-
PASA_GEN_2400149_R	CATCAACTGAACGCTGAGGA	binding EGF-like domains*
NCBI_GNO_2000166_F	ATGCTGTCACAGGAGAGGCT	ATP-dependent RNA helicase*
NCBI_GNO_2000166_R	TTCAGATGCGATAAGCGATG	

Descriptions of DEGs are referred from wFleaBase (*), uniprot (**), interpro (***).
